# Supplementary material for: An increase in reactive oxygen species underlies neonatal cerebellum repair
Source: bioRxiv. 2024 Oct 17:2024.10.14.618368. Preprint. [Version 1] doi: 10.1101/2024.10.14.618368 (PMC11507802; doi:10.1101/2024.10.14.618368)
Supplement: Supplement 1 [file NIHPP2024.10.14.618368v1-supplement-1.pdf]

bioRxiv preprint doi: https://doi.org/10.1101/2024.10.14.618368; this version posted October 17, 2024. The copyright holder for this preprint (which was not certified by peer review) is the author/funder, who has granted bioRxiv a license to display the preprint in perpetuity. It is made available under aCC-BY 4.0 International license.

**A**

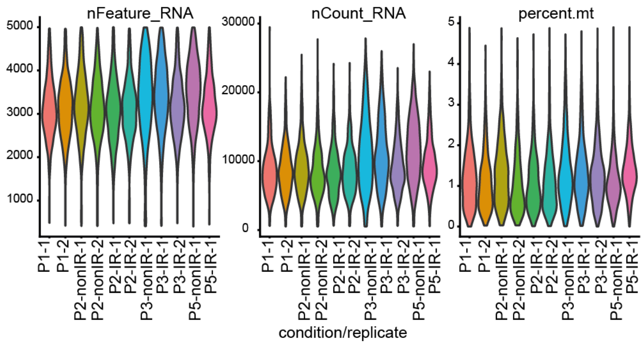


**B**

| | P1 | P2 | | P3 | | P5 | |
|---|---|---|---|---|---|---|---|
| | NON-IR | NON-IR | IR | NON-IR | IR | NON-IR | IR |
| Replicate 1 | 598 | 564 | 1085 | 1293 | 896 | 866 | 2407 |
| Replicate 2 | 643 | 826 | 880 | - | 1330 | - | - |
| Total | 1241 | 1390 | 1965 | 1293 | 2226 | 866 | 2407 |

**C**

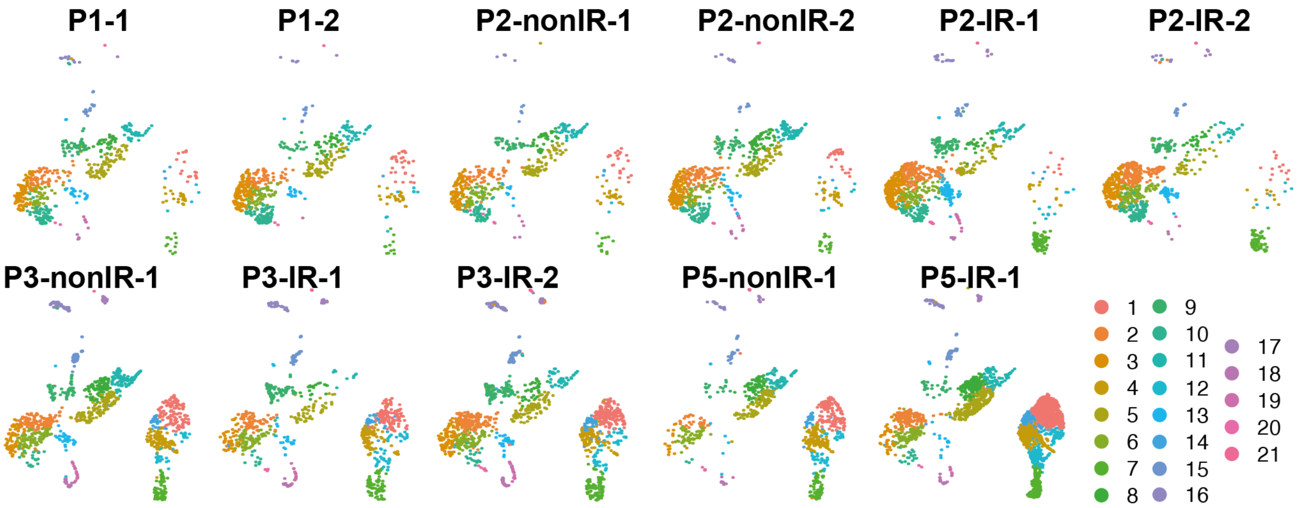


**Supplementary Figure 1. scRNA-seq quality metrics and number of cells sequenced in each condition and biological replicate.**

**(A)** Violin plots showing the number of features, RNA and percent mitochondrial RNA count across the biological replicates of the scRNA-seq data set after filtering the bad quality cells (cells were filtered out where number of detected genes was ≤ 1500, the number of detected transcripts was ≥ 40,000 and mitochondrial gene percentage ≥ 5%).

**(B)** Number of cells from each replicate and condition used for downstream analyses after filtering.

**(C)** UMAPs showing the distribution of cells across different clusters based on the samples.

bioRxiv preprint doi: https://doi.org/10.1101/2024.10.14.618368; this version posted October 17, 2024. The copyright holder for this preprint (which was not certified by peer review) is the author/funder, who has granted bioRxiv a license to display the preprint in perpetuity. It is made available under aCC-BY 4.0 International license.

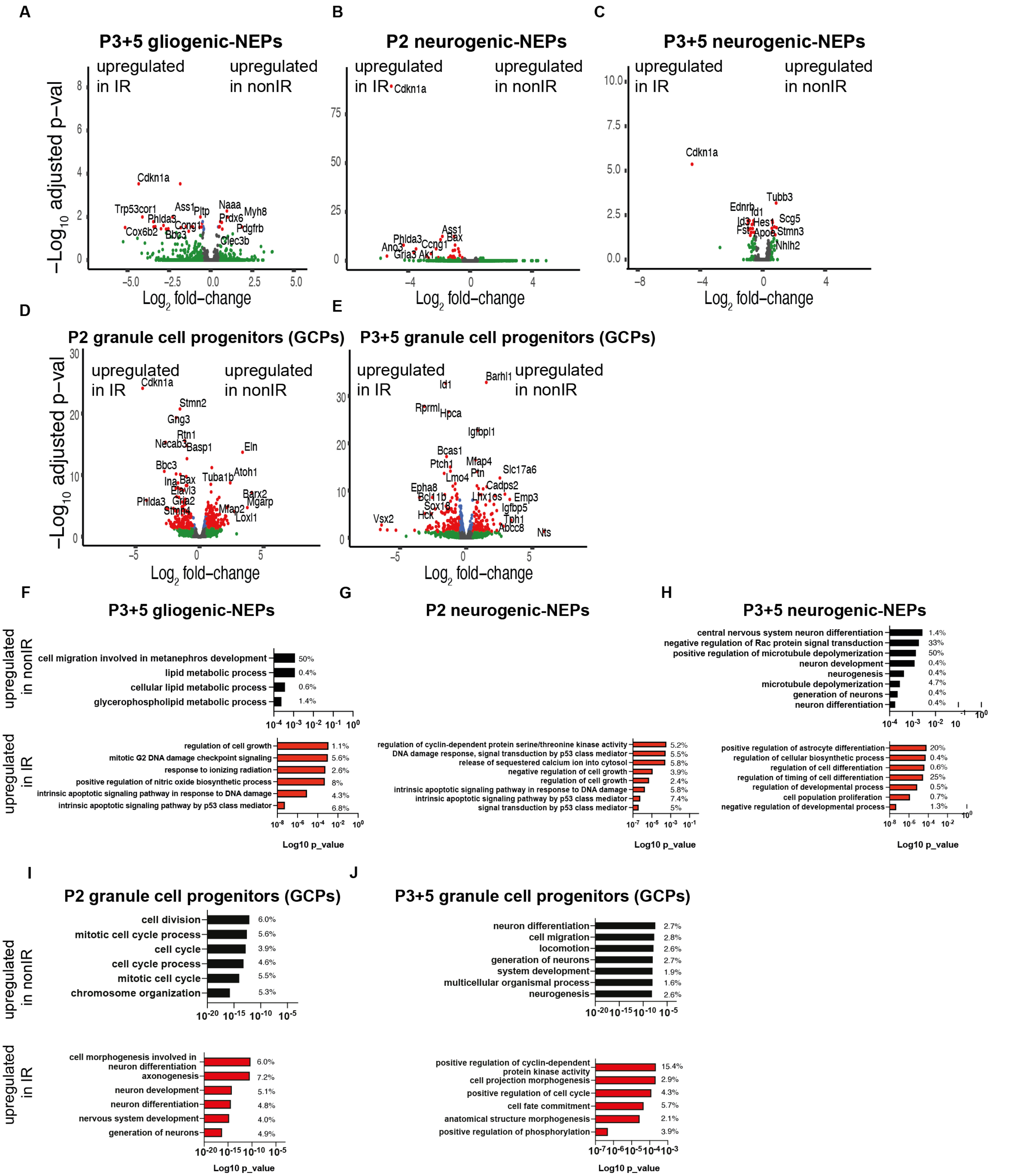


**Supplementary Figure 2: Injury induces distinct transcriptional changes in NEP subtypes and GCPs during adaptive reprograming.**

**(A-E)** Volcano plot showing differentially expressed genes in the P3+5 gliogenic-NEPs (A), P2 and P3+5 neurogenic NEPs (B, C) and P2 or P3+5 GCPs (D, E) (red: adjusted p-value≤0.05, $log_2$fold-change=1, Table S2).

**(F-J)** Top GO terms associated with differentially expressed genes in the P3+5 gliogenic-NEPs (F), P2 or P3+5 neurogenic NEPs (G, H) and P2 or P3+5 GCPs (I, J) that were either upregulated in nonIR (top panel) or IR (bottom panel) (p-value≤0.05).

bioRxiv preprint doi: https://doi.org/10.1101/2024.10.14.618368; this version posted October 17, 2024. The copyright holder for this preprint (which was not certified by peer review) is the author/funder, who has granted bioRxiv a license to display the preprint in perpetuity. It is made available under aCC-BY 4.0 International license.

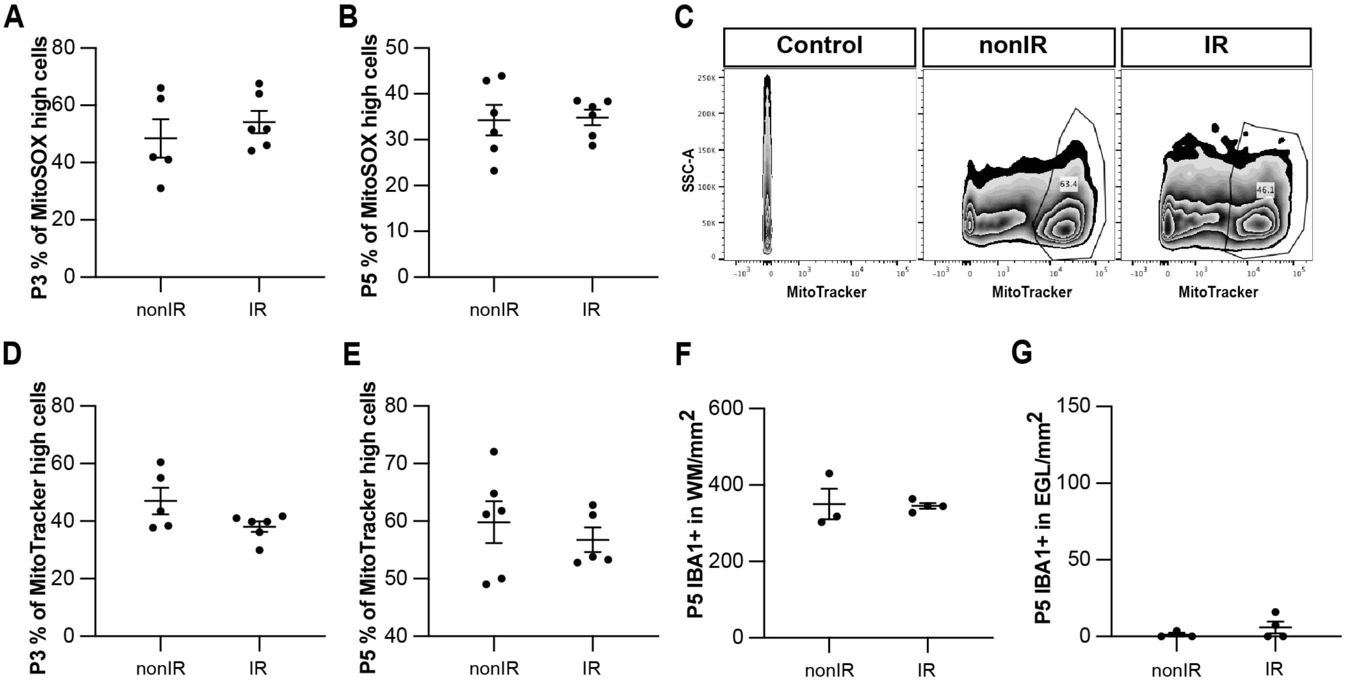


**Supplementary Figure 3: Irradiation of cerebella at P1 results in increased superoxide production and cell death and recruitment of microglia to the EGL that peaks at 24h.**
**(A, B)** Quantification of high MitoSOX expression in nonIR and IR cerebella at P3 (A) and P5 (B).
**(C)** Examples of flow cytometry analysis of mitochondria at P2 from nonIR and IR cerebella using MitoTracker dye. Gating determined the top 90% MitoTracker signal (MitoTracker high cells).
**(D, E)** Quantification of MitoTracker high expression in nonIR and IR cerebella at P3 (D) and P5 (E).
**(F)** Quantification of IBA1+ cell density in the WM at P5 in lobules 3-5 of nonIR and IR mice.
**(G)** Quantification of IBA1+ cell density in the EGL at P5 in lobules 3-5 of nonIR and IR mice.
EGL, External granular layer; WM, White matter; P, postnatal day; nonIR, non-irradiated; IR, irradiated. All statistical significance was determined using an unpaired t-test and data are represented as mean ± SEM.

bioRxiv preprint doi: https://doi.org/10.1101/2024.10.14.618368; this version posted October 17, 2024. The copyright holder for this preprint (which was not certified by peer review) is the author/funder, who has granted bioRxiv a license to display the preprint in perpetuity. It is made available under aCC-BY 4.0 International license.

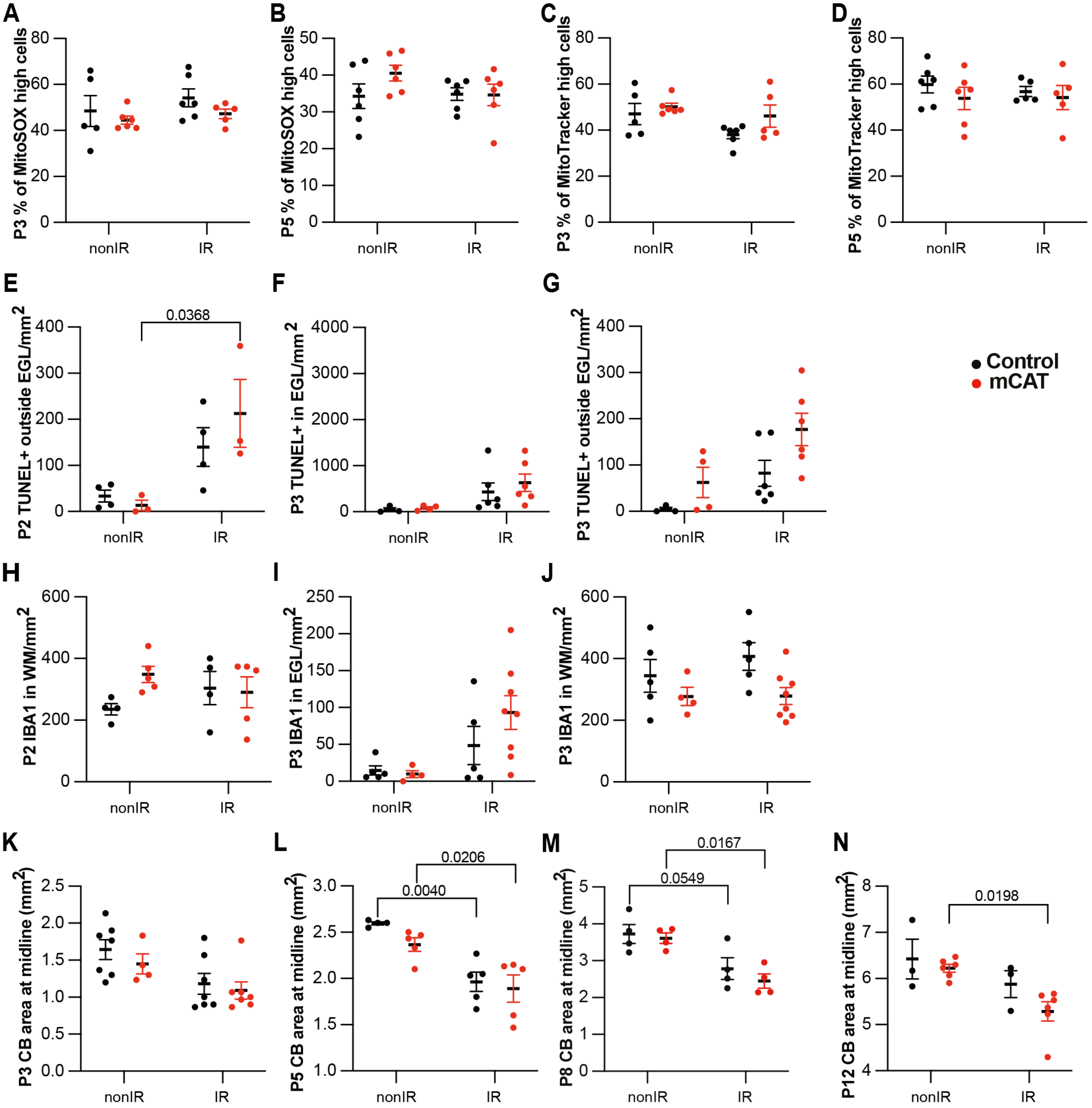


**Supplementary Figure 4: Reduction of ROS impairs adaptive reprogramming and cerebellar repair.**
**(A, B)** Quantification of high MitoSOX expression at P3 (A) and P5 (B) in control and *mCAT/+* cerebella, with and without irradiation at P1.
**(C, D)** Quantification of MitoTracker high expression at P3 (C) and P5 (D) in control and *mCAT/+* cerebella, with and without irradiation at P1.
**(E-G)** Quantification of TUNEL+ cell density outside EGL at P2 (Two-way ANOVA, $F_{(1,10)}$=14.20, p=0.0037) (E), at P3 (G), and in the EGL at P3 (F) in lobules 3-5 of nonIR and IR mice.
**(H-J)** Quantification of IBA1+ cell density in WM at P2 (H), at P3 (J), and in the EGL at P3 (I) in lobules 3-5 of nonIR and IR mice.
(**K-N)** Quantification of cerebellar midsagittal section area in controls and *mCAT/+* nonIR and IR mice at P3 (K), P5 (Two-way ANOVA, $F_{(1,15)}$=28.52, p<0.001) (L), P8 (Two-way ANOVA, $F_{(1,12)}$=21.21, p=0.0006) (M) and P12 (Two-way ANOVA, $F_{(1,14)}$=9.682, p=0.0077) (N).
EGL, External granular layer; WM, White matter; P, postnatal day; nonIR, non-irradiated; IR, irradiated. Significant *Tukey's post hoc* multiple comparison tests are shown in the figures and data are represented as mean ± SEM.

bioRxiv preprint doi: https://doi.org/10.1101/2024.10.14.618368; this version posted October 17, 2024. The copyright holder for this preprint (which was not certified by peer review) is the author/funder, who has granted bioRxiv a license to display the preprint in perpetuity. It is made available under aCC-BY 4.0 International license.

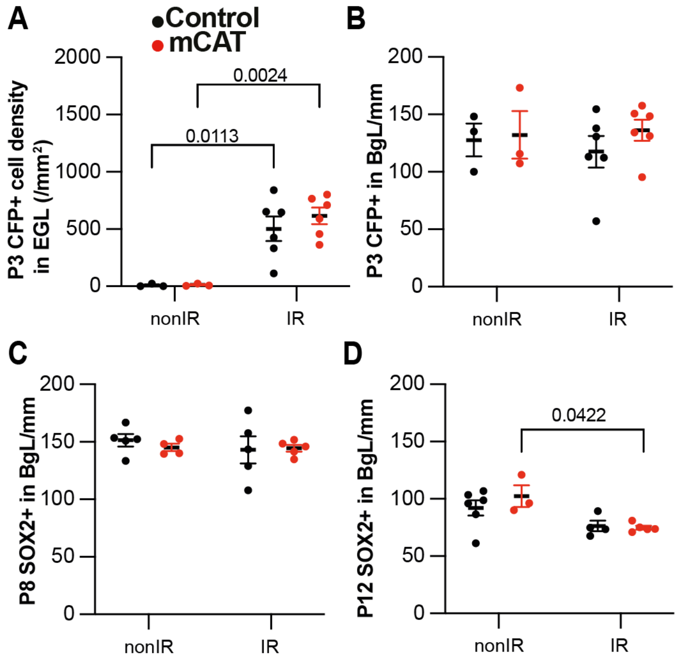


**Supplementary Figure 5: Reduced ROS impairs expansion of BgL-NEPs and their migration to the EGL after injury.**

**(A)** Quantification of CFP+ cell density in the EGL at P3 in *Nes-Cfp/+* control or *Nes-Cfp/+ mCAT* mutant nonIR and IR mice. (Two-way ANOVA, $F_{(1,14)}=33.77$, $p<0.0001$).

**(B)** Quantification of CFP+ cell normalized on BgL length at P5 in *Nes-Cfp/+* control or *Nes-Cfp/+ mCAT* mutant nonIR and IR mice.

**(C, D)** Quantification of SOX2+ NEP cell density on BgL length at P8 (C) and P12 (Two-way ANOVA, $F_{(1,12)}=12.50$, $p=0.0033$) (D) in control or *mCAT* mutant nonIR and IR mice.

EGL, External granular layer; BgL, Bergmann glia Layer; P, postnatal day; nonIR, non-irradiated; IR, irradiated. Significant *Tukey's post hoc* multiple comparison tests are shown in the figures and data are represented as mean ± SEM.

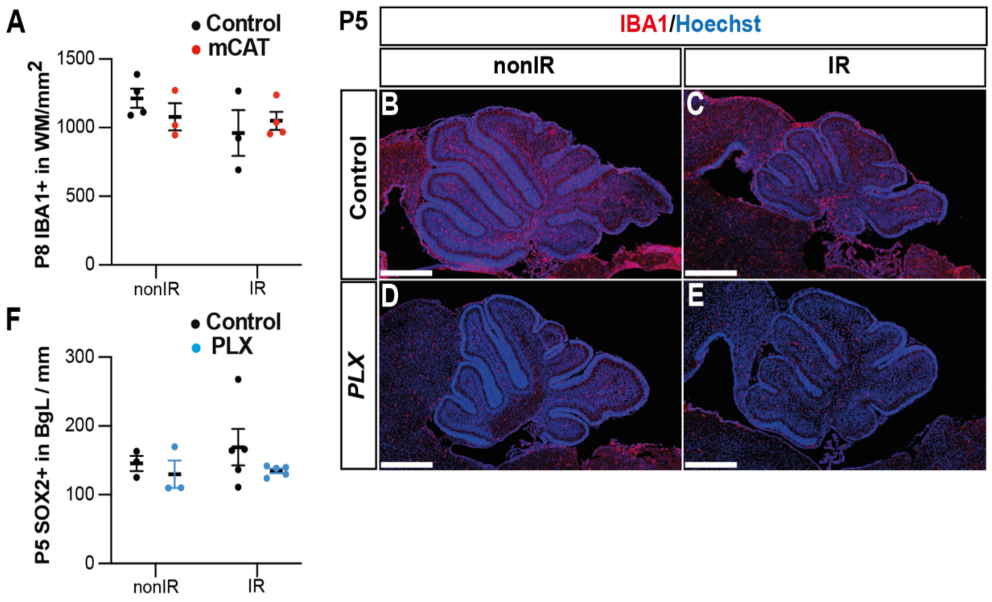


**Supplementary Figure 6: Microglia promote recruitment of NEPs to the EGL during cerebellar adaptive reprogramming after injury.**

**(A)** Quantification of IBA1+ cell density in the WM at P8 in control or *mCAT* mutant nonIR and IR mice.

**(B-E)** Immunostaining of medial sagittal cerebellar sections at P5 showing expression of IBA1 (red) in mice treated with PLX5622 or control DMSO, with or without irradiation.

(F) Quantification of SOX2+ NEP cell density in the BgL at P5 in control or PLX treated nonIR and IR mice.

BgL, Bergmann glia Layer; WM, White Matter; P, postnatal day; nonIR, non-irradiated; IR, irradiated. Scale bar: 500 µm. Data are represented as mean ± SEM.

bioRxiv preprint doi: https://doi.org/10.1101/2024.10.14.618368; this version posted October 17, 2024. The copyright holder for this preprint (which was not certified by peer review) is the author/funder, who has granted bioRxiv a license to display the preprint in perpetuity. It is made available under aCC-BY 4.0 International license.

## Supplementary Tables

| Antigen | Species | Concentration | References | Source |
|---|---|---|---|---|
| Catalase | Rabbit | 1 to 100 | 01-05-030000 | Athens Research & Technology |
| GFAP | Chicken | 1 to 500 | ab4674 | Abcam |
| GFP | Rat | 1 to 1000 | 440484 | Nacalai Tesque |
| IBA1 | Rabbit | 1 to 500 | 019-19741 | Wako Chemicals |
| SOX2 | Goat | 1 to 500 | AF2018 | R&D System |

**Table S1**: List of antibodies and related information.

**Table S2.** Marker genes expressed by cluster in scRNA-seq dataset (irradiated at P1 (IR; P2, P3, P5) or non-irradiated (nonIR; P1, P2, P3, P5). pct1: % cells in a cluster that express the gene; pct2: % cells that express the gene outside the given cluster.

**Table S3.** Pseudobulk differential expression analysis between nonIR and IR gliogenic-NEPs (*Hopx+,* clusters 2, 3, 6, 10), neurogenic-NEPs (*Ascl1+,* clusters 5, 8, 11) and GCPs (*Atoh1+,* clusters 1,4,7,12,14) at P2, or at P3 and P5 (P3+5).

**Table S4.** GO Term analyses of differentially expressed genes (Table S3) of nonIR and IR gliogenic-NEPs (*Hopx+,* clusters 2, 3, 6, 10), neurogenic-NEPs (*Ascl1+,* clusters 5, 8, 11) and GCPs (*Atoh1+,* clusters 1,4,7,12,14) at P2, or at P3 and P5 (P3+5).

**Table S5**. Differentially open peaks at P2 identified by bulk ATAC-seq from nonIR and IR NEPs.

**Table S6**. Motif analysis of regions with increased accessibility in IR NEPs compared to the nonIR at P2.
